# Global metabolomics reveals metabolic dysregulation in ischemic retinopathy

**DOI:** 10.1007/s11306-015-0877-5

**Published:** 2015-11-18

**Authors:** Liliana P. Paris, Caroline H. Johnson, Edith Aguilar, Yoshihiko Usui, Kevin Cho, Lihn T. Hoang, Daniel Feitelberg, H. Paul Benton, Peter D. Westenskow, Toshihide Kurihara, Jennifer Trombley, Kinya Tsubota, Shunichiro Ueda, Yoshihiro Wakabayashi, Gary J. Patti, Julijana Ivanisevic, Gary Siuzdak, Martin Friedlander

**Affiliations:** Department of Cell and Molecular Biology, The Scripps Research Institute, MB 28, 10550 North Torrey Pines Road, La Jolla, CA 92037 USA; Scripps Center for Metabolomics and Mass Spectrometry, The Scripps Research Institute, 10550 North Torrey Pines Road, La Jolla, CA 92037 USA; Department of Ophthalmology, Tokyo Medical University, 6-7-1 Nishishinjuku, Shinjuku-ku, Tokyo, 160-0023 Japan; Departments of Chemistry, Genetics, and Medicine, Washington University School of Medicine, St. Louis, MO 63110 USA; The Lowy Medical Research Institute, 3030 N. Torrey Pines Court, La Jolla, CA 92037 USA

**Keywords:** Untargeted metabolomics, Pathway enrichment analysis, Proliferative diabetic retinopathy, Arginine metabolism

## Abstract

Proliferative diabetic retinopathy (PDR) is the most severe form of diabetic retinopathy and, along with diabetic macular edema, is responsible for the majority of blindness in adults below the age of 65. Therapeutic strategies for PDR are ineffective at curtailing disease progression in all cases; however a deeper understanding of the ocular metabolic landscape in PDR through metabolomic analysis may offer new therapeutic targets. Here, global and targeted mass spectrometry-based metabolomics were used to investigate metabolism. Initial analyses on vitreous humor from patients with PDR (n = 9) and non-diabetic controls (n = 11) revealed an increase of arginine and acylcarnitine metabolism in PDR. The oxygen-induced-retinopathy (OIR) mouse model, which exhibits comparable pathological manifestations to human PDR, revealed similar increases of arginine and other metabolites in the urea cycle, as well as downregulation of purine metabolism. We validated our findings by targeted multiple reaction monitoring and through the analysis of a second set of patient samples [PDR (n = 11) and non-diabetic controls (n = 20)]. These results confirmed a predominant and consistent increase in proline in both the OIR mouse model and vitreous samples from patients with PDR, suggesting that over activity in the arginine-to-proline pathway could be used as a therapeutic target in diabetic retinopathy.

**Electronic supplementary material:**

The online version of this article (doi:10.1007/s11306-015-0877-5) contains supplementary material, which is available to authorized users.

## Introduction

Diabetic retinopathy (DR) is the leading cause of vision loss in adults aged 20–64 years (Ding and Wong 2012). Meta-analysis of large-scale studies has shown that approximately one-third of the diabetic population will develop DR to some extent and approximately one-third of those (or 10 % of the whole diabetic population) will progress to its vision-threatening stages, PDR and diabetic macular edema (Zheng et al. 2012). Prevention strategies focusing on early screening and optimization of metabolic control have been implemented, and have improved the outlook for patients in many countries. Nevertheless they have proved to be insufficient on their own to fully, and efficiently, arrest DR progression towards late stage disease (Das et al. 2015). Proliferative diabetic retinopathy (PDR), the most advanced stage of DR, is especially concerning. It is associated with macular edema and can lead to hemorrhage and tractional retinal detachment, which constitute leading causes of blindness in diabetic patients (Fong et al. 1999). In addition, the current therapeutic strategies (laser photocoagulation and anti-VEGF intraocular injections) are not able to curtail disease progression in a sustainable and effective manner for every patient. Moreover, concerns regarding safety issues with anti-VEGF agents have been raised at the ocular (choroidal vasculature and photoreceptors) and systemic levels (renal and cardiovascular effects) (Cheung et al. 2014; Georgalas et al. 2014; Lains et al. 2014).

Incomplete understanding of the pathophysiology of DR is exacerbated by the absence of an in vivo model that fully recapitulates the disease. As rodent models of diabetes do not spontaneously develop pre-retinal neovascularization, the oxygen-induced-retinopathy (OIR) mouse model is frequently used in studies of neovascular eye disease such as PDR (Robinson et al. 2012; Stahl et al. 2010). The OIR model resembles retinopathy of prematurity by developing regions of vascular obliteration and pathological neovascularization after a 5-day exposure to a hyperoxic environment, which arrests physiologic retinal vascular development. These retinal findings are also seen in human PDR.

Metabolites are the biological products of genomic and proteomic perturbations, and also result from environmental (e.g., diet, toxins) and microbial influences. Global (untargeted) metabolomic analysis is the unbiased survey of all metabolites within a sample, and can reveal biologically relevant changes within a system. Previous metabolomic studies performed in pre-diabetic and type 2 diabetic patients revealed that amino-acid and lipid concentration changes can be used as biomarkers for identifying patients at risk, and also for monitoring disease progression and therapeutic efficacy (Roberts et al. 2014). Indeed, unraveling major metabolic changes in the vitreous of PDR patients has the potential to reveal novel targets for the development of more effective therapeutic strategies to treat patients with diabetic eye disease. Our global and highly sensitive targeted mass spectrometry (MS)-based metabolomic workflows allow for a comprehensive coverage of the metabolome (Ivanisevic et al. 2013). Although reproducibility is a concern in metabolomics, especially when translation into the clinic is being considered, the high number of clinical and mouse samples available in our study has allowed for multiple opportunities for validation, enhancing the robustness and reliability of the data.

**Fig. 1:**
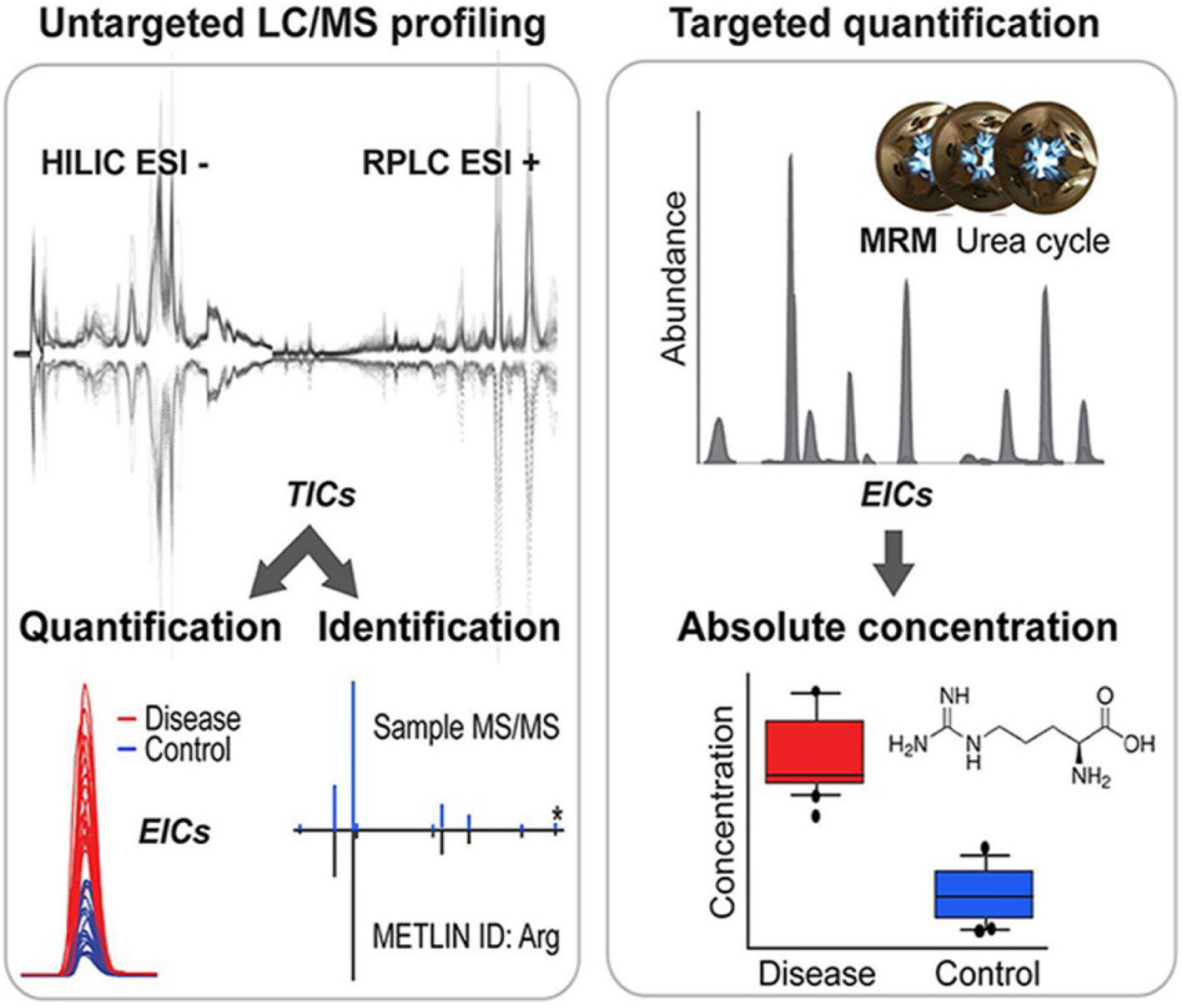
Metabolomic workflow. Samples initially undergo untargeted quadrupole time-of-flight mass spectrometry (QTOF–MS) metabolomics by hydrophilic interaction and reversed-phase liquid chromatography (HILIC and RPLC) to obtain a comprehensive coverage of the metabolome. Metabolites are identified using the statistical software XCMS Online and the METLIN database. Tandem MS is carried out to verify the metabolite identification. The metabolites of interest are further validated through multiple reaction monitoring by triple quadrupole (QqQ)-MS with authentic standards, and absolute concentrations obtained

In this study, a global MS-based approach was used to generate and validate a metabolomic profile of vitreous samples from two separate patient sample sets (control and patients with PDR) as well as eyes from the OIR mouse model. Simultaneously, we reiterated the validity of the OIR mouse model for therapeutic studies regarding PDR by demonstrating shared metabolic dysregulation with the human disease, despite differences in the pathological trigger. An overview of our metabolomic workflow can be seen in Fig. 1 which highlights our untargeted metabolomic approach with validation by targeted selected reaction monitoring (SRM) analysis. Both reversed phase liquid chromatography (RPLC) and hydrophilic interaction liquid chromatography (HILIC) analysis were used to obtain a comprehensive qualitative coverage of the metabolome with subsequent quantitative validation. The novel metabolic findings reported here will pave the way towards identification of new disease biomarkers, discovery of new druggable targets and development of more effective therapeutic algorithms.

## Materials and methods

### Patients

Twenty type 2 diabetic patients with PDR and 31 non-diabetic controls with epiretinal membrane or macular hole participated in this study and underwent vitreo-retinal surgery at the ophthalmology department of Tokyo Medical University. Meta-data for these individuals can be seen in Table S1.

### Collection of vitreous samples

All patients underwent standard pars plana vitrectomy under local anesthesia. Vitrectomy was conducted using a 25-gauge 3-port system and with a high-speed vitreous cutter (2500 cycle/min). Phacoemulsification and aspiration were performed simultaneously in patients with cataracts, for which an acrylic foldable intraocular lens was placed in the capsular bag. Undiluted vitreous samples (0.5–1 mL) were aspirated from the mid-vitreous under standardized conditions at the beginning of the surgery, transferred to sterile tubes, frozen immediately (within 15 s) and stored in liquid nitrogen (including during shipping) until analysis, this ensured quenching of the metabolism. Vitreous samples were collected from a total of 20 patients with PDR (samples with vitreous hemorrhage were excluded from the analysis) and 31 non-diabetic control patients undergoing surgery for removal of epiretinal membranes and repair of macular holes.

### Animals and the oxygen induced retinopathy (OIR) model

OIR was induced in C57BL/6 mice according to the protocol by Smith et al. (1994). Briefly, 7 day-old C57BL/6 mice were placed in 75 % oxygen for 5 days and were returned to ambient oxygen up to another 5 day-period. Whole eyes were collected immediately after death at twelve (P12, n = 3), fourteen (P14, n = 3) and seventeen (P17, n = 4) days after birth, frozen directly in liquid nitrogen and stored at −80 °C until analysis. Age matched C57BL/6 mice raised in ambient oxygen (normoxia) were used as controls (n = 5/time point).

## Sample extraction for mass spectrometry-based metabolomics

### Human vitreous

Acetone (400 μl) was added to 1.5 ml glass high recovery vials (Agilent Technologies, Santa Clara, CA, USA) containing 100 μl of human vitreous and was vortexed for 30 s. The samples were then placed in liquid nitrogen for 1 min, thawed for 5 min and sonicated for 15 min. This cycle was repeated two more times before the samples were stored overnight at −20 °C. After storage, the samples were centrifuged for 15 min at 13,000 rpm at 4 °C in 1.5 ml microcentrifuge tubes. The supernatant was transferred to 1.5 ml glass vials and stored at −20 °C until later use. The pellet was resuspended in 400 μl methanol/water (80:20 v/v), vortexed for 30 s, sonicated for 15 min and the supernatant pooled with the supernatants previously collected. The samples were stored at −20 °C for 1 h, centrifuged for 15 min at 13,000 rpm, 4 °C, and the supernatants dried in a speedvac. All samples were resuspended in 100 μl acetonitrile:methanol:isopropanol (40:40:10), sonicated for 20 min, centrifuged for 15 min at 13,000 rpm, 4 °C, and transferred to autosampler vials for storage at −80 °C until use (Patti et al. 2012).

### Mouse whole eye

For the analysis of the mouse model, whole eyes were taken (two per sample) and homogenized in 400 μl methanol/water (80:20 v/v) with 1 mm glass beads (Biospec, Bartlesville, OK, USA) in 1.5 ml glass vials. A minilys homogenizer (Bertin Technologies, Montigny le bretonneux, France) was used for 30 s at 3000 rpm. The samples were sonicated for 15 min and stored overnight at −20 °C. The samples were centrifuged at 13,000 rpm for 15 min at 4 °C. The supernatant was transferred to 1.5 ml glass vials and stored at −20 °C until later use. The pellet was resuspended in 600 μl acetone and homogenized again for 10 s, and stored at −20 °C overnight. The samples were centrifuged at 13,000 rpm for 15 min at 4 °C and the supernatant pooled with previously retained supernatant. The samples were dried down in a speedvac and resuspended in 80 μl acetonitrile/water (50/50 v/v), sonicated for 5 min, centrifuged for 15 min at 13,000 rpm, 4 °C, and transferred to autosampler vials for storage at −80 °C until use.

To note two different resuspension solvent mixtures were used for the different eye tissues. These methods were optimized for maximal recovery of both hydrophobic and hydrophilic metabolites.

### Global metabolomic analysis

Analyses were performed using a high performance liquid chromatography (HPLC) system (1200 series, Agilent Technologies) coupled to a 6538 UHD quadrupole time-of-flight (Q-TOF) mass spectrometer (Agilent Technologies). Samples were injected (8 μl) onto either a Zorbax C18, 5 µm, 150 mm × 0.5 mm I. D. column (Agilent Technologies) for RPLC analysis, or a Luna Aminopropyl, 3 µm, 150 mm × 1.0 mm I.D. column (Phenomenex, Torrance, CA) for HILIC analysis. Pooled samples were injected every three samples and a blank after every samples for quality control. The standard mobile phase for RPLC was A = 0.1 % formic acid in water and B = 0.1 % formic acid in acetonitrile in electrospray ionization (ESI) positive mode. For HILIC the mobile phase was A = 20 mM ammonium acetate and 20 mM ammonium hydroxide in 95 % water and B = 95 % acetonitrile in ESI negative mode. The linear gradient elution from 100 % B (0–5 min) to 100 % A (50–55 min) was applied in HILIC at a flow rate of 50 µL/min and from 100 % A (0–5 min) to 100 % B (50–55 min) in RPLC at a flow rate of 20 µL/min. A 10 min post-run was applied for HILIC, to insure column re-equilibration and maintain reproducibility.

ESI source conditions were set as followings: gas temperature 325 °C, drying gas 5 L/min, nebulizer 15 psi, fragmentor 120 V, skimmer 65 V, and capillary voltage 4000 V or −4000 V in ESI positive or ESI negative modes, respectively. The instrument was set to acquire over the *m/z* range 60–1000, with the MS acquisition rate of 2.4 spectra/s. For the MS/MS of selected precursors the default isolation width was set as medium (4 Da), with a MS acquisition rate at 2.63 spectra/s and MS/MS acquisition at 2.63 spectra/s. The collision energy was fixed at 20 eV.

LC/MS data were processed using XCMS Online (Tautenhahn et al. 2012). Features were listed in a feature list table and as an interactive cloud plot, containing their integrated intensities (extracted ion chromatographic peak areas) observed fold changes across the two sample groups, and p-values for each sample (Patti et al. 2013). The default XCMS parameter set for HPLC–UHD–QTOFMS was used with tolerance for database search set to 30 ppm. Integration of METLIN to XCMS Online allowed for putative identification of metabolites. Identifications were then made by comparing retention times and tandem MS fragmentation patterns to the sample and a standard compound (purchased from Sigma Aldrich, St.Louis, MO, USA).

### Tandem MS experiments

Were carried out with the collision energy set to 20 eV and caused the fragmentation of the metabolites into a number of fragments specific for the metabolite. This fragmentation pattern combined with the retention time comparison to a standard allows for accurate identification. The full datasets are available as public shares on XCMS Online.

### Targeted metabolomic analysis

Samples (8 μL) were injected onto a Luna Aminopropyl column or Zorbax C18 using the same LC conditions as described for the global analysis. SRM triple quadrupole mass spectrometry (Agilent 6410 QqQ-MS) were used with quantifier and qualifier transitions for each metabolite as seen in Table S2. ESI source conditions were set as followings: gas temperature 325 °C, drying gas 5 L/min, nebulizer 15 psi, fragmentor 120 V, skimmer 65 V, and capillary voltage 4000 or −4000 V in ESI positive or ESI negative modes, respectively. The instrument was set to acquire over the *m/z* range 60–1000, with the MS acquisition rate of 1.67 spectra/s. For the MS/MS of selected precursors the default isolation width was set as medium (4 Da), with a MS acquisition rate at 1.67 spectra/s and MS/MS acquisition at 1.67 spectra/s. The collision energy was fixed at 20 eV.

### Statistical analysis

Statistical analysis of the metabolomic data was performed by XCMS (employing a two-sample Welch’s *t* test with unequal variances). The Student’s *t* test for unpaired data was used to compare control to OIR mice using the software Prism (where p < 0.05 was considered statistically significant). Wilcoxon rank sum test was used to compare non-diabetic control to PDR samples.

## Results and discussion

### Global metabolomics revealed a clear distinction between PDR and control vitreous human samples

**Fig. 2:**
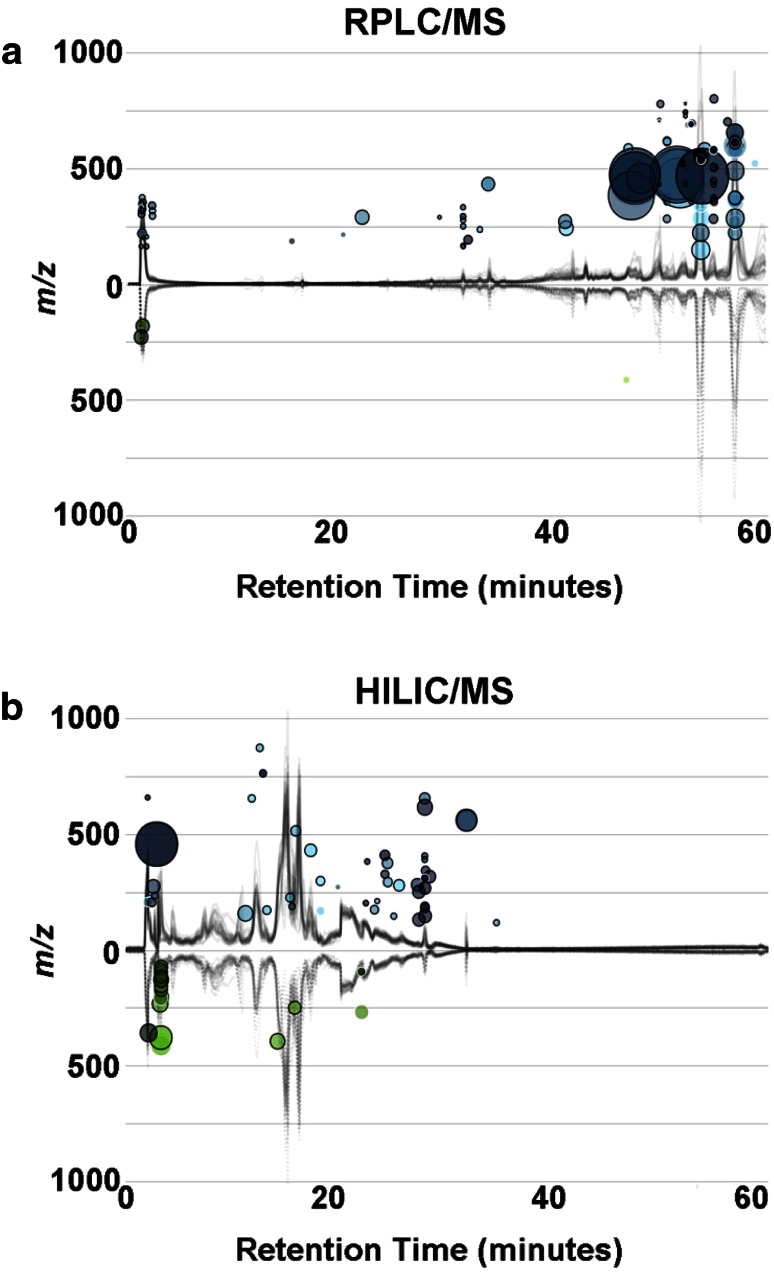
Global liquid chromatography quadrupole time-of-flight mass spectrometry (LC-QTOFMS) metabolomics. Cloud plots generated by XCMS Online showing dysregulated features between control and PDR samples (two-tailed Mann–Whitney test) for **a** RPLC–MS analysis, control (n = 11), PDR (n = 9) top plot and **b** HILIC–MS analysis, control (n = 11), PDR (n = 7), lower plot. Total ion chromatograms (TICs) for each sample can be seen on the plot; features whose intensity are increased in PDR vitreous are shown on the upper part of the plot as blue circles and features whose intensity decreases are shown on the bottom part of the plot as green circles. Larger and brighter circles (features) correspond to larger fold changes and lower p-values respectively

Although the presence of metabolic dysfunction has been widely explored in other tissues in diabetic conditions, little is known about what happens in the eye in PDR. Global metabolomic analysis by RPLC–MS and HILIC–MS provided a comprehensive coverage of the non-polar and polar metabolome, respectively. The analyses revealed clear dysregulation (meaning differential regulation) in the first set of human vitreous samples between non-diabetic controls (n = 11) and patients with PDR (n = 9). RPLC–MS analysis revealed 106 features that were significantly dysregulated (p < 0.01, fold change >2) from a total of 3165 aligned features (Fig. 2a). Of these features, a number were adducts and fragment ions. A q-value threshold of <0.05 was used to remove any p-values (up to a 95 % confidence) that could have been false positives (Storey 2002; Storey and Tibshirani 2003). The metabolites that were positively identified by tandem MS with comparison to authentic standards included the following metabolites increased in PDR samples: octanoylcarnitine (fold change 5.3, p = 0.005, q = 0.01) and propionylcarnitine (fold change 2.1, p = 0.007, q = 0.02). Other carnitines mined for in the feature tables that had higher p-values than 0.01 were similarly increased in PDR; hexanoylcarnitine (fold change 4.4, p = 0.005, q = 0.01), acetylcarnitine (fold change 1.5, p = 0.012, q = 0.02), palmitoylcarnitine (fold change 3.8, p = 0.038, q = 0.04) and elaidic/vaccenylcarnitine (fold change 4.9, p = 0.035, q = 0.04). HILIC–MS analysis between non-diabetes controls (n = 11) and patients with PDR (n = 7) revealed 61 dysregulated features from a total of 1962 total aligned features (Fig. 2b). The features that were positively identified and increased in the PDR samples included allantoin (fold change 4.0, p = 0.006, q = 0.03), glutamate (fold change 3.2, p = 0.0002, q = 0.008), lysine (fold change 1.7, p = 0.004, q = 0.03), and arginine (fold change 2.1, p = 0.005, q = 0.03). A second set of samples were obtained from non-diabetic control (n = 20) and PDR (n = 11) patients, which allowed us to observe the specificity of the metabolites. The RPLC–MS metabolomic analysis did not show dysregulated acylcarnitines and only revealed 30 dysregulated features, from a total of 6834 aligned features, this low number of dysregulated features (0.4 %) shows that with a p-value threshold of 0.01 these are most likely random. These results do show a variation in the RPLC–MS results between the first and second sample sets. It is possible that this comes from the analytical platform used. However, humans have high interindividual variation, and carnitine metabolism in particular is dependent on diet as well as other factors; Carnitines also have low specificity for one disease, therefore it was not surprising not to see a validation of carnitine dysregulation in the 2nd set of patient samples. In any case all the metabolites observed between both data sets were targeted for SRM analysis to further validate the metabolite presence, the data is shown below. The HILIC–MS analysis was more reproducible between the two sets of patient samples and showed 129 dysregulated features from a total of 7827 aligned features. Again increases in allantoin (fold change 1.9, p = 0.001, q = 0.03), glutamate (fold change 2.8, p = 0.0001, q = 0.007), lysine (fold change 2.1, p = 0.001, q = 0.02), and arginine (fold change 1.6, p = 0.002, q = 0.04) were revealed in patient PDR samples. In this 2nd set of samples HILIC–MS also showed a number of metabolites downregulated in the PDR samples, N-acetylaspartate (fold change 2.3, p = 0.0006, q = 0.02; supplementary material 2), iditol (fold change 2.0, p = 0.002, q = 0.03), glycerate (fold change 1.8, p = 0.002, q = 0.04) and N-acetylglutamate (fold change 1.8, p = 0.0004, q = 0.02; supplementary material 2). These metabolites were confirmed through tandem MS to standards. In addition, these downregulated metabolites were mined for in the data from the first set of samples but they were not dysregulated.

**Fig. 3:**
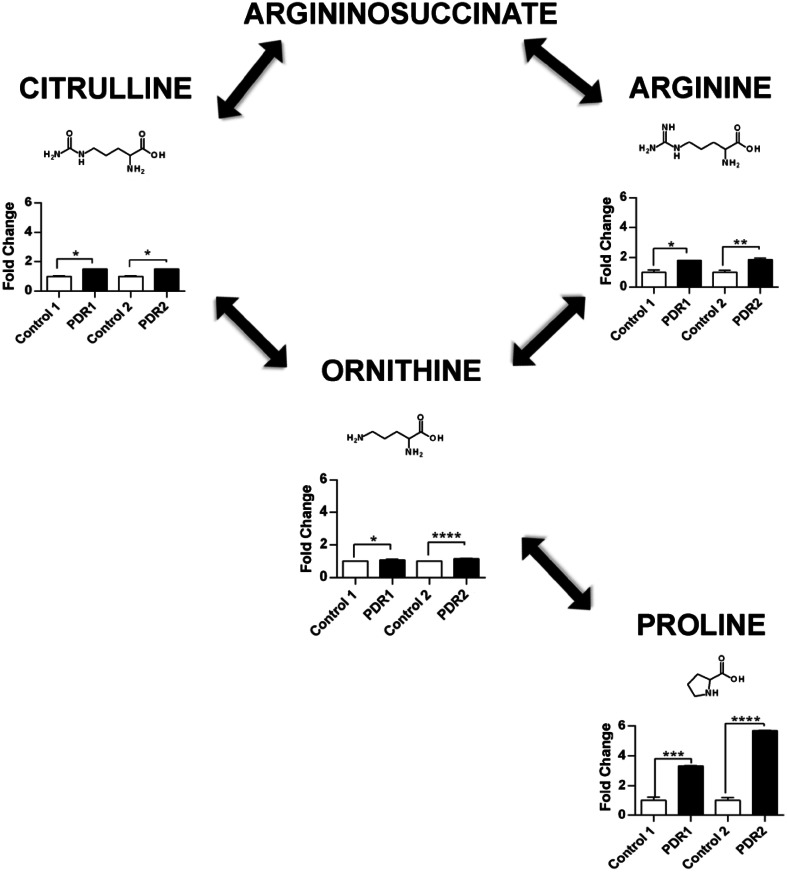
Significant metabolic perturbations identified in human PDR. The arginine-to-proline pathway shows the highest number of metabolic perturbations in this disease, fold changes of each metabolite in PDR samples are shown compared to control *=p ≤ 0.05, **=p ≤ 0.01, ***=p ≤ 0.001,****=p ≤ 0.0001, error bars are standard deviation

Having two sets of human samples from non-diabetic controls and patients with PDR has been invaluable to show the specificity of these biomarkers to this disease. Some metabolites were perturbed in both, while some specific to just one set. Untargeted metabolomics by QTOF–MS can reveal thousands of features, many novel to the disease, however specificity can be more challenging with human sample analysis due to interindividual variation providing lower p-values. In addition, QTOF–MS analysis is not as quantitative as targeted triple quadrupole (QqQ)-MS analysis due to coeluting ions and detector saturation, and when fold changes are subtle (in the range of 1–2.5) it is difficult to assess specificity. Thus, we next sought to validate our findings using targeted analysis to quantify the concentrations of these metabolites. As well as targeting the metabolites dysregulated in both sample sets, we expanded the targeted analysis to include metabolites from related metabolic pathways, to determine the biological relevance of our findings. These pathways included those related to acylcarnitine and amino acid metabolism (aconitate, fumarate, succinate, glutamine, pantothenate, proline, citrate), nitrogen disposal (citrulline, ornithine) and purine metabolism-related oxidative stress (AMP, ATP, adenosine, inosine, IMP, hypoxanthine, xanthine). Targeted analysis was thus carried out using authentic standards to obtain accurate fold changes of the metabolites in the extracted samples (Fig. 3; Table 1). It was confirmed that arginine and allantoin, metabolites seen in both sets of samples, were increased in PDR, however lysine and glutamate were not changed. Some of the metabolites targeted were below the limit of detection in the samples, but an increase in octanoylcarnitine could also be confirmed in both sample sets, and further dysregulation was seen in proline, citrulline, methionine and ornithine along with decanoylcarnitine, which were all significantly increased in the samples from PDR patients (Fig. 3; Table 1). A summary of the targeted analysis for both sets of human samples can be seen in Table 1.

**Table 1:** Validated list of metabolites changed in human vitreous samples when comparing controls to patients with PDR and in mouse eyes from normoxia and OIR models at P17

Metabolite	Human PDR				OIR P17 mouse	
	First set		Second set			
	Fold change	p-value	Fold change	p-value	Fold change	p-value
Methionine^a^	1.7	0.0387	3.0	0.0002	1.1	0.6436
Allantoin^a^	2.5	0.0003	1.7	0.0081	1.4	0.2349
Decanoylcarnitine^a^	1.7	0.0028	1.4	0.0054	Below limit of detection	
Arginine^a,b^	1.8	0.0387	1.9	0.0081	2.2	0.0109
Proline^a,b^	3.3	0.0003	5.7	<0.0001	5.0	0.0002
Citrulline^a,b^	1.5	0.0201	1.5	0.0211	2.0	0.0003
Ornithine^a,b^	1.1	0.0346	1.2	<0.0001	1.3	0.0084
Octanoylcarnitine^a,b^	2.2	0.0200	1.7	0.0005	3.0	0.0004
Lysine^b^	1.3	0.0573	1.1	0.2383	1.5	0.0024
Succinate^b^	1.4	0.6180	1.3	0.8580	−1.6	0.0226
Pantothenate^b^	Below limit of detection				1.7	0.0175
AMP^b^	Below limit of detection				−1.4	0.0477
Hypoxanthine^b^	1.4	0.0573	1.4	0.2542	−3.4	<0.0001
Xanthine^b^	Below limit of detection				−1.9	0.0017
Inosine^b^	Below limit of detection				−2.8	<0.0001
Propionylcarnitine^b^	Below limit of detection				86.4	0.0480
Acetylcarnitine^b^	Below limit of detection				2.0	<0.0001

^a^Statistically significant dysregulation in Human PDR

^b^Statistically significant dysregulation in the OIR mouse

The concomitant increase of various amino acids in the vitreous of human PDR patients suggests a compromise in the metabolic capacity of the retina and raises concerns about potential amino acid toxicity and indiscriminate use of amino acid supplements by diabetic patients.

### The OIR mouse model mimics human PDR metabolic dysregulation, and progresses over the hypoxic phase

Since the OIR mouse model spontaneously develops several pathological retinal features that are also observed in PDR, (e.g., retinal ischemia, pre-retinal neovascularization and profound neurodegeneration), global and targeted metabolomic analyses were performed on ocular samples from these mice to identify and validate their metabolic profile at the time of maximal pre-retinal neovascularization (17 days of age, P17). We compared whole eyes extracted from OIR mice and controls raised in ambient oxygen and found that metabolites involved in the arginine pathway/urea cycle (arginine, proline, citrulline and ornithine) and in beta oxidation (octanoylcarnitine) were similarly dysregulated to that observed in clinical samples from PDR patients (Table 1).

In the OIR mouse model methionine and allantoin were not dysregulated, but lysine, pantothenate and succinate were increased, when compared to controls. Furthermore, a downregulation in the purine metabolism pathway was seen in the OIR model [adenosine monophosphate (AMP), inosine, hypoxanthine and xanthine] (Table 1).

To further understand how these metabolic alterations develop over the hypoxic period (from P12 until P17) in the OIR mouse, global metabolomics was carried out on ocular samples collected at P12, P14 and P17. They were compared to those of age-matched controls raised in ambient oxygen normoxia (NOX mice). Pantothenate was increased with fold changes of 1.6 (p = 0.0154, q = 0.3746, P12) and 2.0 (p = 0.009, q = 0.1909, P14). Proline, arginine and lysine were increased from P14 onwards with fold changes of 3.1 (p = 0.0000009, q = 0.0006), 2.3 (p = 0.0164, q = 0.2356), and 2.7 (p = 0.0017, q = 0.1175), respectively, while at P17 they were increased 3.1 (p = 0.0049, q = 0.1537), 3.1 (p = 0.009, q = 0.1596) and 2.1 (p = 0.0398, q = 0.1764) respectively. The q-values are out of the ideal threshold of 0.05, however the p-values are very low and indicate a trend for these metabolites.

Of note, different extraction protocols were used to extract the vitreous humor and whole eyes samples. The vitreous humor extraction was optimized by trying combinations of extraction solvents; methanol:acetonitrile (1:1 v/v), an acetone-based extraction with methanol:water (80:20 v/v), adapted from previously published protocols (Patti et al. 2012). Mechanical breakdown techniques were also compared, bead homogenization vs. vortexing with three freeze/thawing cycles, as were the type of reconstitution solvents. The extraction protocol which enabled the largest protein precipitation with the largest metabolite recovery required acetone followed by methanol:water (80:20 v/v) with vortexing/freeze thawing, and reconstitution in acetonitrile:methanol:isopropanol (40:40:10 v/v/v); the full method is detailed in the methods section. For the whole eyes, a similar protocol was used, however bead homogenization was initially carried out in methanol:water (80:20 v/v), as the tissue was tough to break down by vortexing/freeze thawing only. Acetone could only be added after homogenization as it would have caused further dehydration of the tissue. The optimal reconstitution solvent was acetonitrile/water (50/50 v/v). The same method was not used for both types of sample extraction as it would not have been possible to recover as many metabolites from these very different matrices. Furthermore even though the injection solvents were different, metabolite retention time and peak shapes were reproducible between the analyses.

### Arginine metabolism and ammonia detoxification are similarly dysregulated in human PDR and in the OIR mouse

**Fig. 4:**
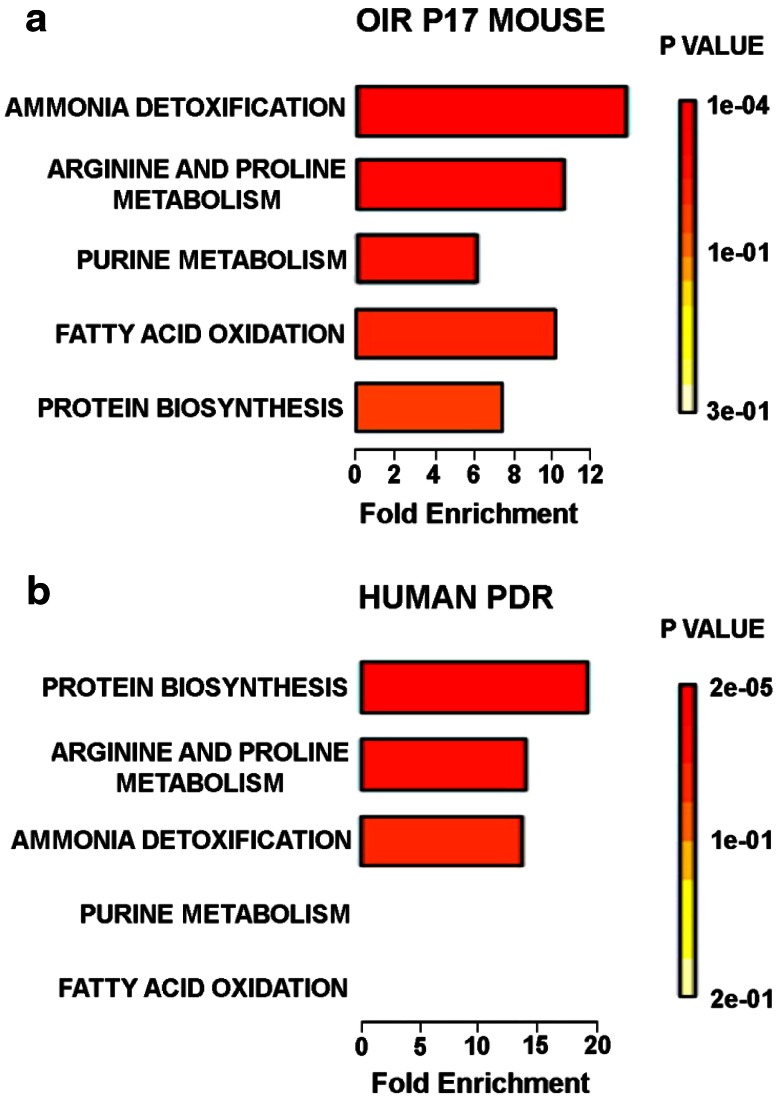
Metabolite Set Enrichment Analysis. Arginine metabolism and urea cycle (ammonia disposal) pathways are the most significantly affected both in **a** OIR P17 mouse (top panel; 4/26; FDR = 0.0184 and 4/20; FDR = 0.0126) and in the **b** Human PDR (lower panel; 4 dysregulated features out of 26; False discovery rate (FDR) p-value = 0.00353 and 3/20; FDR = 0.0249, respectively)

Pathway enrichment analysis was performed for the metabolites dysregulated in the OIR mouse and human PDR samples using the MetaboAnalyst program (Xia et al. 2009). The analysis revealed that arginine metabolism and ammonia detoxification (urea cycle) were two of the most perturbed pathways in both species for the conditions under study, being dysregulated to a similar magnitude (Fig. 4a, b). This adds to a growing body of evidence that suggests that Mueller glial cell metabolism is particularly compromised in DR and that this potentially disrupts the neurovascular crosstalk within the retina, thus promoting disease progression (Bringmann et al. 2006; Metea and Newman 2006).

Arginine metabolism has been widely studied in other diabetic tissues as well as in diabetic rodent models that recapitulate only the early stages of DR (Narayanan et al. 2013, 2014; Patel et al. 2013). Two different pathways in the retina can metabolize arginine: the arginase pathway that produces ornithine and urea, and the nitric oxide synthase (NOS) pathway, which generates citrulline and NO. Current knowledge suggests that the pathological features observed in diabetic rodent model retinas are caused by over activity of the enzyme arginase II (Patel et al. 2013). This causes a shortage of arginine for the NOS pathway, resulting in NO sub-availability, endothelial cell dysfunction, and, consequently, impaired vasodilation. It also causes NOS uncoupling with subsequent increased production of oxygen and nitrogen reactive species that contribute to further retinal damage (Narayanan et al. 2013).

In vitreous samples from PDR patients, simultaneous increases of metabolites involved in both the arginase and the NOS pathway were observed (arginine, ornithine, proline and citrulline; Table 1); however the most pronounced perturbations were seen in proline levels, therefore investigating how and why this happens in the diabetic eye may provide important clues for better understanding the pathophysiology of DR. Ornithine and proline can be generated from arginine via the arginase pathway whereas citrulline is produced via the NOS pathway. Overexpression and excessive activity of the arginase pathway in the diabetic rodent retina has been implicated in vascular endothelial cell dysfunction, via reduced activity in the NOS pathway (Patel et al. 2013) with subsequent increase in formation of peroxynitrite, polyamines and proline, which induce cellular proliferation and fibrosis. Arginase overactivity has also been described in the OIR model, where it contributes to hyperoxia-induced retinal neurodegeneration, via upregulation of polyamine synthesis, and retinal microvascular dropout via increased oxidative stress (Narayanan et al. 2011, 2014).

Fatty acid oxidation was also disturbed both in the OIR mouse and in human PDR as a result of dysregulated acylcarnitines (Table 1; Fig. 4a, b); however, the fatty acid oxidation pathway was not sufficiently enriched in the pathway enrichment analyst for human PDR due to an incomplete reference metabolite dataset for this pathway (Fig. 4b). In the OIR mouse eye, there was an additional compromise in purine metabolism revealed by a significant downregulation of AMP, inosine, hypoxanthine and xanthine (Fig. 4a).

## Conclusion

DR is the most common cause of blindness in adults below the age of 65 due to development of diabetic macular edema and PDR. Currently, there are no validated biomarkers to reliably monitor disease status and no effective therapeutic strategies to arrest disease progression in every patient with PDR. In addition there is no diabetic rodent model that spontaneously recapitulates PDR’s pathological features. Here, we generated a metabolomic profile for PDR in diabetic patients that revealed significant impairment to amino acid, acylcarnitine and purine metabolism. This profile was validated in a second set of patient samples, and was also observed in the non-diabetic OIR mouse (that develops similar retinal pathological features), suggesting it as a promising model for studies focused on PDR.

## Electronic supplementary material

Supplementary material 1 (DOCX 35 kb)

Supplementary material 2 (DOCX 165 kb)

## Abbreviations

PDR: Proliferative diabetic retinopathy
DR: Diabetic retinopathy
DN: Diabetic nephropathy
DNP: Diabetic neuropathy
PC: Photocoagulation
HM: Hand movements
IOP: Intraocular pressure
BCVA: Best corrected visual acuity
NOX: Normoxia
OIR: Oxygen induced retinopathy
