# Supplementary material for: Global metabolomics reveals metabolic dysregulation in ischemic retinopathy
Source: Metabolomics. 2015 Nov 18;12:15. doi: 10.1007/s11306-015-0877-5 (PMC4651979; doi:10.1007/s11306-015-0877-5)
Supplement: Supplementary file 1 — Supplementary material 1 (DOCX 35 kb) [file 11306_2015_877_MOESM1_ESM.docx]

**Supplementary Information**

**Table S1 - Clinical characteristics of the patients with PDR.** In order to contextualize the metabolic perturbations in PDR, the 20 study subjects with PDR (8 M; 12 F) underwent comprehensive clinical evaluation including a detailed medical history and laboratory work-up. This information is present in **Table. S1**. All subjects had type 2 diabetes with an average duration of 11.3 years (since diagnosis) and an average HbA1c of 7.43 %. Their best corrected visual acuity (BCVA) ranged from hand motion (HM) to 0.6 (20/33), intraocular pressure ranged from 7 mmHg to 24 mmHg (average of 14.59 mmHg) and 50% of the patients had undergone laser photocoagulation that had been complete in 90% of the cases. Regarding other diabetes-related systemic complications, 35% did not have any reported complications other than DR; in 15% there was no available information; and 50% had other systemic complications. Within the latter, 30% had isolated diabetic nephropathy, 20% had isolated diabetic neuropathy and 50% had both nephropathy and neuropathy.

| Gender/age | BCVA | PC | IOP mmHg | DM | Years since DM diagnosis | HbA1c % | Other DM complications |
| --- | --- | --- | --- | --- | --- | --- | --- |
| 54 F | HM | Complete | 10 | 2 | 10 | 10.3 | DNP, DN |
| 67 F | 0.02 | Complete | 13 | 2 | 15 | 9.2 | DN |
| 51 M | HM | Complete | 15 | 2 | 2 | 10.9 | DN |
| 69 M | 0.03 | Complete | 20 | 2 | 24 | 6.9 | DNP, DN |
| 42 F | 0.04 | 0 | 24 | 2 | 8 | 6.8 | 0 |
| 42 F | 0.03 | 0 | 18 | 2 | 8 | 6.8 | 0 |
| 61 M | HM | Complete | 16 | 2 | 6 | 6.2 | DN |
| 75 M | 0.5 | 0 | 14 | 2 | 2 | 7.6 | 0 |
| 62 M | 0.2 | 0 | 14 | 2 | 24 | 6.7 | DNP |
| 61 M | 0.6 | Complete | 11 | 2 | 20 | 6.6 | 0 |
| 40 M | 0.03 | Complete | 10.7 | 2 | 20 | 5.3 | DNP, DN |
| 65 F | 0.07 | 0 | 16 | 2 | 9 | 7.5 | 0 |
| 48 M | 0.4 | 0 | 15 | 2 | 15 | 9.1 | DNP |
| 35 M | HM | 0 | 15 | 2 | 10 | 8.5 | DNP, DN |
| 65 F | 0.08 | Partial | 11 | 2 | 10 | 6.8 | 0 |
| 68 M | 0.04 | 0 | 10 | 2 | 24 | 5.7 | DNP, DN |
| 58 M | HM | Complete | 7 | 2 | 15 | 6.9 | 0 |
| 56 F | 0.4 | Complete | 17 | 2 | 2 | 5.3 | N/A |
| 68 F | 0.03 | 0 | 16 | 2 | 1 | 8.7 | N/A |
| 71M | 0.01 | 0 | 19 | 2 | 1 | 6.8 | N/A |

DN - diabetic nephropathy; DNP - diabetic neuropathy; PC – photocoagulation; HM - hand movements; IOP - intraocular pressure; BCVA – best corrected visual acuity; N/A – not available.

**Table S2. List of metabolites targeted by multiple reaction monitoring.**

| Standard Compound | ESI Mode | Precursor Ion | Quantifier Ion | Fragmentor Voltage (V) | Collision Energy (V) | Qualifier Ion | Fragmentor Voltage (V) | Collision Energy (V) |
| --- | --- | --- | --- | --- | --- | --- | --- | --- |
| Oleoylcarnitine | Positive | 426.4 | 85 | 137 | 27 | 57 | 137 | 63 |
| Myristoylcarnitine | Positive | 372.3 | 85 | 137 | 27 | 57 | 137 | 47 |
| Decanoylcarnitine | Positive | 316.2 | 85 | 14 | 19 | 57 | 14 | 47 |
| Octanoylcarnitine | Positive | 288.2 | 85 | 17 | 19 | 57 | 17 | 39 |
| Hexanoylcarnitine | Positive | 260.2 | 85 | 137 | 43 | 60 | 137 | 15 |
| Propionylcarnitine | Positive | 218.1 | 85 | 131 | 15 | 158.9 | 131 | 7 |
| Acetylcarnitine | Positive | 204.1 | 85 | 131 | 15 | 43 | 131 | 47 |
| Acetyl-CoA | Positive | 810 | 303 | 135 | 28 |  |  |  |
| Adenosine | Positive | 268.1 | 136 | 131 | 15 | 118.9 | 131 | 47 |
| Citrulline | Positive | 176.1 | 158.9 | 98 | 7 | 70 | 98 | 23 |
| Methionine | Positive | 150 | 104 | 77 | 7 | 56.1 | 77 | 31 |
| Lysine | Positive | 147 | 130.1 | 77 | 7 | 84.1 | 77 | 15 |
| Proline | Positive | 116 | 70.1 | 86 | 15 | 43.1 | 86 | 43 |
| ATP | Negative | 506 | 158.9 | 156 | 30 | 79 | 156 | 75 |
| PRPP | Negative | 388.9 | 291 | 95 | 6 | 176.9 | 95 | 14 |
| IMP | Negative | 347 | 135.1 | 109 | 26 | 92.1 | 109 | 54 |
| AMP | Negative | 346 | 134 | 127 | 34 | 79 | 127 | 70 |
| Inosine | Negative | 267 | 135 | 129 | 17 | 108 | 129 | 37 |
| Ribose-5-phosphate | Negative | 229 | 139 | 89 | 6 | 97 | 89 | 6 |
| Pantothenate | Negative | 218 | 146.1 | 61 | 10 | 88 | 61 | 8 |
| Citrate | Negative | 191 | 111 | 77 | 6 | 87 | 77 | 14 |
| Arginine | Negative | 173.1 | 156.1 | 61 | 4 | 131.1 | 61 | 10 |
| Cis-Aconitate | Negative | 173 | 129 | 76 | 4 | 85 | 76 | 8 |
| Aminoadipate | Negative | 160.1 | 142 | 70 | 8 | 116 | 70 | 10 |
| Allantoin | Negative | 157 | 114 | 70 | 9 | 97 | 70 | 9 |
| Xanthine | Negative | 151 | 108 | 114 | 13 | 42.1 | 114 | 25 |
| Glutamate | Negative | 146 | 128.1 | 80 | 6 | 102 | 80 | 2 |
| Glutamine | Negative | 145.1 | 127.1 | 83 | 6 | 109.1 | 83 | 10 |
| α-ketoglutarate | Negative | 145 | 101 | 40 | 5 | 57 | 40 | 5 |
| Hypoxanthine | Negative | 135 | 92 | 111 | 13 | 65 | 111 | 29 |
| Malate | Negative | 133 | 115 | 50 | 10 | 71 | 50 | 10 |
| Oxaloacetate | Negative | 131 | 87 | 55 | 7 |  |  |  |
| Succinate | Negative | 117 | 73 | 62 | 9 |  |  |  |
| Fumarate | Negative | 115 | 71 | 60 | 5 |  |  |  |
| Lactate | Negative | 89 | 43.2 | 55 | 12 |  |  |  |
| Pyruvate | Negative | 87 | 43 | 55 | 3 |  |  |  |

ESI; Electrospray Ionization
