# Supplementary material for: Global metabolomics reveals metabolic dysregulation in ischemic retinopathy
Source: Metabolomics. 2015 Nov 18;12:15. doi: 10.1007/s11306-015-0877-5 (PMC4651979; doi:10.1007/s11306-015-0877-5)
Supplement: Supplementary file 2 — Supplementary material 2 (DOCX 165 kb) [file 11306_2015_877_MOESM2_ESM.docx]

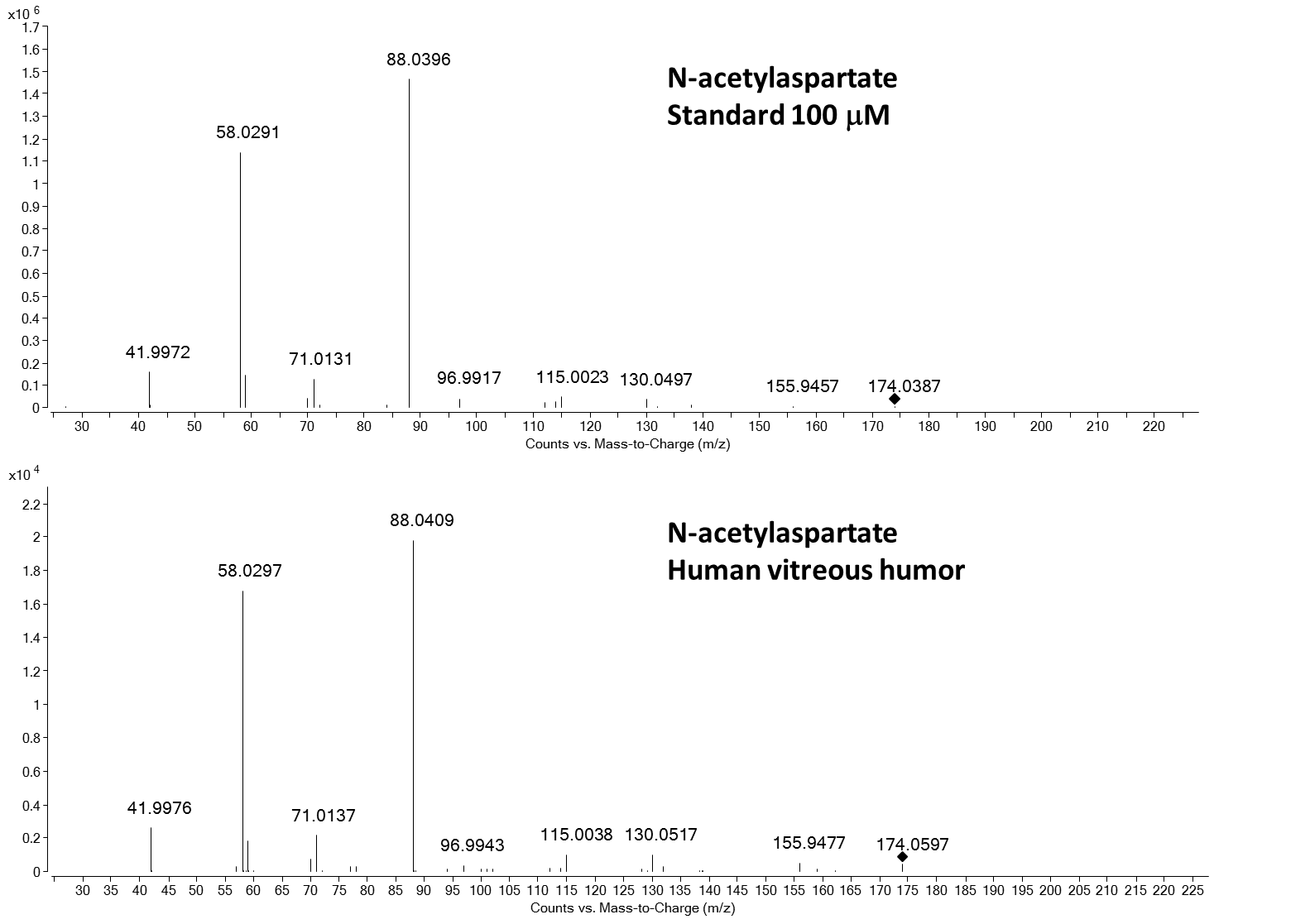


Tandem mass spectra of m/z 174.0416 in electrospray negative ionization mode. Top panel N-acetylaspartate standard, lower panel ion in pooled vitreous humor control samples.


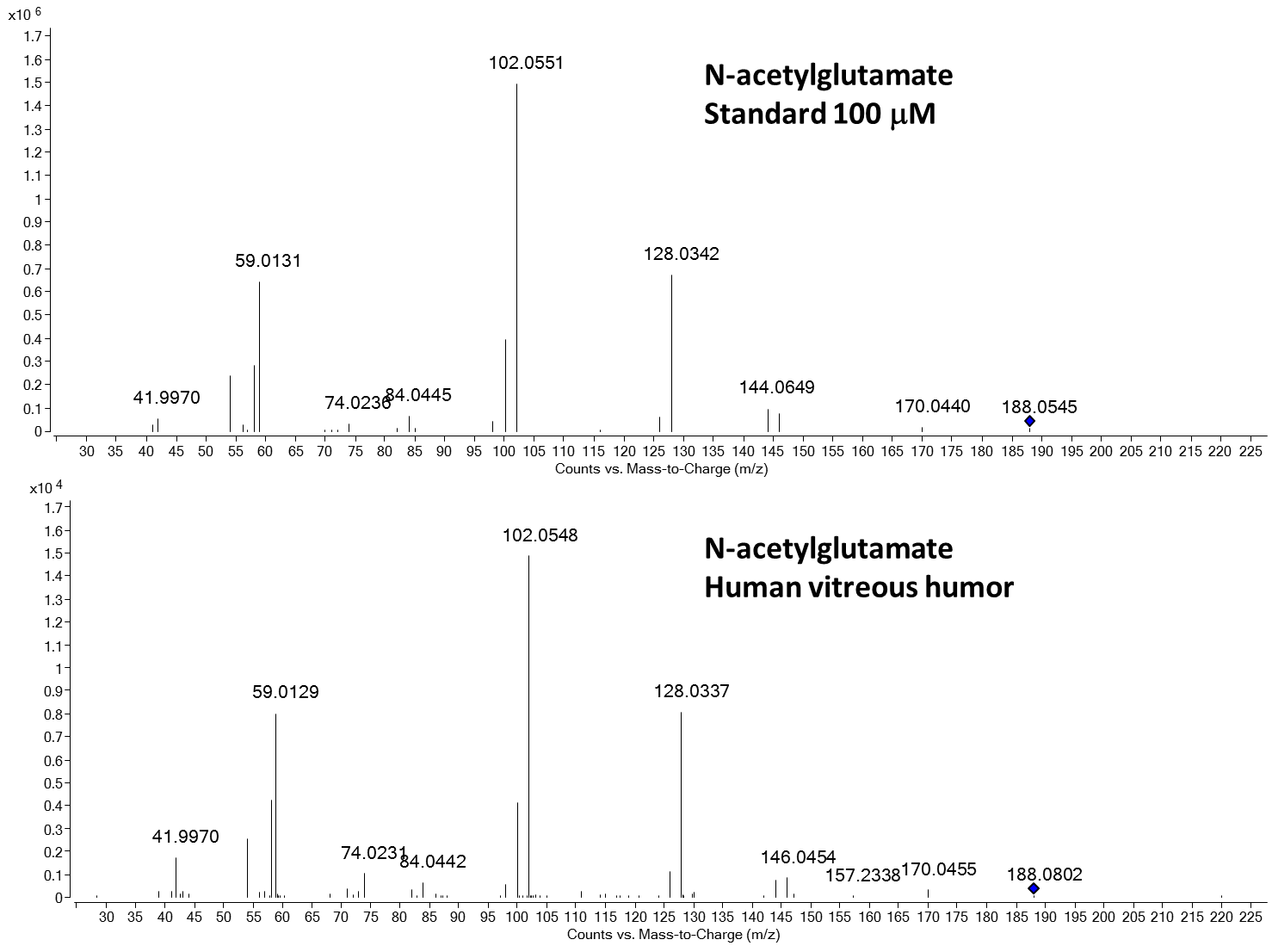


Tandem mass spectra of m/z 188.0572 in electrospray negative ionization mode. Top panel N-acetylglutamate standard, lower panel ion in pooled vitreous humor control samples.
